# Controlled inter-state switching between quantized conductance states in resistive devices for multilevel memory†

**DOI:** 10.1039/c9ra00726a

**Published:** 2019-03-25

**Authors:** Sweety Deswal, Rupali R. Malode, Ashok Kumar, Ajeet Kumar

**Affiliations:** Academy of Scientific and Innovative Research, CSIR-National Physical Laboratory Campus Dr. K. S. Krishnan Marg New Delhi 110012 India kumarajeet@nplindia.org; CSIR-National Physical Laboratory Dr. K. S. Krishnan Marg New Delhi 110012 India; Maulana Azad National Institute of Technology Bhopal Madhya Pradesh 462003 India

## Abstract

A detailed understanding of quantization conductance (QC), the correlation with resistive switching phenomena and controlled manipulation of quantized states is crucial for realizing atomic-scale multilevel memory elements. Here, we demonstrate highly stable and reproducible quantized conductance states (QC-states) in Al/niobium oxide/Pt resistive switching devices. Three levels of control over the QC-states, required for multilevel quantized state memories, like, switching ON to different quantized states, switching OFF from quantized states, and controlled inter-state switching among one QC state to another has been demonstrated by imposing limiting conditions of stop-voltage and current compliance. The well-defined multiple QC states along with a working principle for switching among various states show promise for implementation of multilevel memory devices.

## Abstract 2

Stable quantized conductance states were achieved and controllably switched to higher or to lower conductance states for multilevel memory.

## Graphical abstract

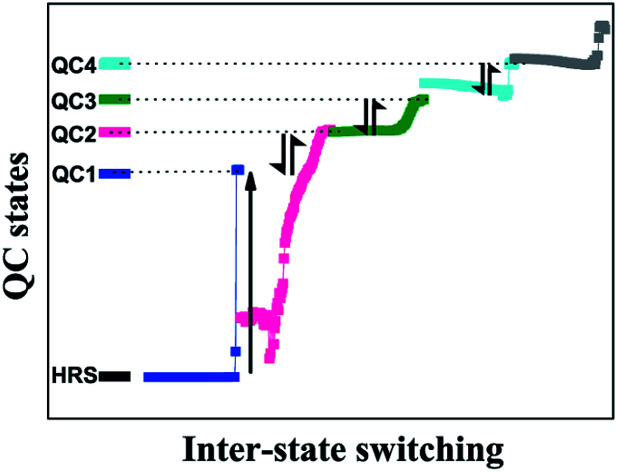

Driven by the demand for improved computing capability, the semiconductor industry is following the extension of Moore's law which says that the density of transistors in an integrated circuit doubles every two years. However the current technology, charge based flash memory, has reached its limit of miniaturization.^1,2^ Also, all existing devices are limited to two stable memory states (*i.e.*, “0” and “1”). Increasing the number of stable states, from bi-stability to multi-stability, will be an effective method for producing high-density and efficient memory devices.

As one for the most promising candidates for future non-volatile memories, resistive random access memory (ReRAM) with simple two-terminal sandwiched structured devices exhibit attractive performances due to their scalability down to the atomic level, CMOS compatibility, low-power consumption, and high-speed features.^3,4^ It has been proposed that the multiple stable states available in the resistive switches can be used for multilevel storage for ultrahigh density memories.^5^ Existence of stable multistates has been demonstrated in resistive switching,^6–14^ ferroelectric^15–20^ and phase change^21–25^ memory devices. Atomic point contact based QC observed in resistive switching devices has also been demonstrated for memory applications.^26–31^ However, controlled manipulation of multiple stable states for potential application in multilevel memory is yet to be achieved.

Several kinds of control over stable QC-states in a resistive switching device are required to achieve multilevel quantized state memories. These particular kinds of devices have not been fully explored, partly because of the lack of appropriate materials and lack of design & working principles. Many groups have demonstrated quantization in several ReRAM^32–36^ as well as in atomic switch^29,37–39^ devices. The conditions to achieve different quantized states either with current compliance^40,41^ or with stop voltage^37,40^ have been reported. Also, there is some understanding about the stability of these states with respect to time.^35,37,42,43^ However, conditions for controlled inter-QC-state switching, essential for multilevel memory, have not been reported.

Here, we demonstrate control over the events of switching ON to different QC-states, switching OFF from QC-states, and inter-QC-state switching in Al/niobium oxide/Pt device. Firstly, stable and reproducible QC-states with integer and half-integer multiples of quantum of conductance (*G*_0_ = 2*e*^2^/*h* ∼77.4 μS) were achieved, indicating formation of well-controlled atomic point contacts in the conducting filaments. Then, the devices were manipulated to exhibit hundreds of different inter-QC-state switching, both in the direction of SET (higher *G*_0_) or RESET (lower *G*_0_) starting from any particular QC-state. The initial and final QC-states, for each switching event, were found to be stable. The device exhibited longer retention times for higher QC-states. Rules for controlled switching are evolved with stop-voltage and current compliance limits during current–voltage (*I*–*V*) measurements. The working principles demonstrated in this work, to achieve QC-states and to induce inter-QC-state switching, is a crucial step towards realization of multilevel memory devices.

*Switching ON to QC-state*: The resistive switching and QC characteristics are demonstrated using *I*–*V* measurements on Al/Nb_2_O_5_/Pt devices in air at room temperature. These devices, in their pristine state, were found in high resistance OFF state (HRS) of the order of ∼10^9^ Ω. Initially, the device was switched to low resistance ON state (LRS) at a forming voltage ∼4 V with current compliance (*I*_c_) of 5 μA, as shown in the inset of Fig. 1a. After forming, with voltage sweeps, the device showed reproducible switching between LRS to HRS (RESET; voltage ∼ −0.4 to −1.2 V) and *vice versa* (SET; voltage ∼1.6–2.5 V), shown as semi-logarithmic *I*–*V* plots in Fig. 1a. These devices show both unipolar as well as bipolar switching characteristics in either polarities of the voltage (ESI Fig. S1†). In our previous work,^34^ unipolar switching behaviour of the Al/Nb_2_O_5_/Pt devices were presented and it was demonstrated that the conducting filament, after making the atomic point contact, grows in thickness atom-by-atom during SET voltage sweep. Here, in this work, conducting filaments were stabilized to achieve various QC-states. During the SET process, the LRS was controlled by applying voltage sweeps with different current compliance values of 100, 200, 300, 400 and 500 μA (Fig. 1b) and different resistance states of 9 kΩ, 6 kΩ, 4 kΩ, 2.9 kΩ and 2.3 kΩ, respectively, were achieved. These resistance states were stable and correspond to quantized conductance states of ∼1.5 *G*_0_, ∼2 *G*_0_, ∼3.5 *G*_0_, ∼4.5 *G*_0_, and ∼5.5 *G*_0_, respectively (Fig. 1c).

**Fig. 1:**
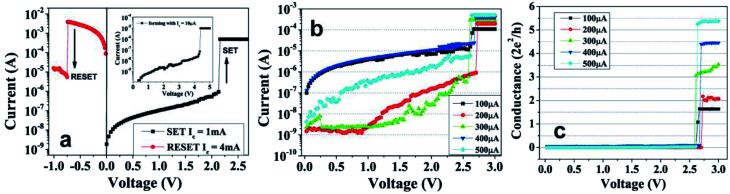
(a) Semi-logarithmic *I*–*V* characteristics of Al/Nb_2_O_5_/Pt device showing bipolar switching with SET and RESET in range of 1.6–2.5 V and –(0.4–1.2 V), respectively. The inset shows the electroformation step of the device. (b) The semi-logarithmic *I*–*V* plots of SET with various current compliances (*I*_c_) values of 100, 200, 300, 400, 500 μA reaching to different LRS levels corresponding to quantized conductance states of ∼1.5 *G*_0_, ∼2 *G*_0_, ∼3.5 *G*_0_, ∼4.5 *G*_0_, and ∼5.5 *G*_0_, respectively. (c) The SET traces of (b) are plotted as conductance *vs.* voltage (*G*–*V*) to show distinguishable quantized LRS states obtained.

QC-states achieved during SET sweeps with different *I*_c_ values were analyzed to determine the state distribution of the device conductance. Fig. 2a–f show that distinct and stable QC-states could be reproducibly achieved by varying *I*_c_ values. Histograms of conductance in the units of *G*_0_ for ∼300 switching cycles performed on an Nb_2_O_5_ device are shown for five different compliance currents upto 500 μA. After each SET event, the conductance state was estimated by applying a read voltage of 100 mV. The data was sorted in the bin size of 0.1 *G*_0_ and respective numbers were counted to plot the conductance histogram, shown in Fig. 2. QC-state of ∼1 *G*_0_ was achieved with 100 μA, where only a single conduction channel allows electron transport through the filament of the resistive switch. With the increase of current compliance, the conductance peak shifted towards higher conductance value. The QC-states of 2 *G*_0_, 3.5 *G*_0_, 4.5 *G*_0_, and 5.5 *G*_0_ were achieved with *I*_c_ of 200 μA, 300 μA, 400 μA, and 500 μA, respectively, as seen in Fig. 2b–e. As higher conductance states are gradually reached, it has been understood that the atomic rearrangements in the point contact allows more number of conduction channels to become available for electron transfer.^31,34^ The histogram with all five sets of each *I*_c_, acquired from ∼300 curves of SET cycles is shown in Fig. 2f. It can be clearly seen that the devices exhibited quantized conductance peaks around integer and half-integer multiples of *G*_0_. Out of several switching cycles, 25 cycles with each *I*_c_ values 100, 200, 300, 400, 500 μA representing the median of the distribution of quantized state as plotted in Fig. 2a–e is shown in Fig. 2g. The well separated memory levels available in our devices meet one of the essential requirements for realizing multilevel ultra high density storage.

**Fig. 2:**
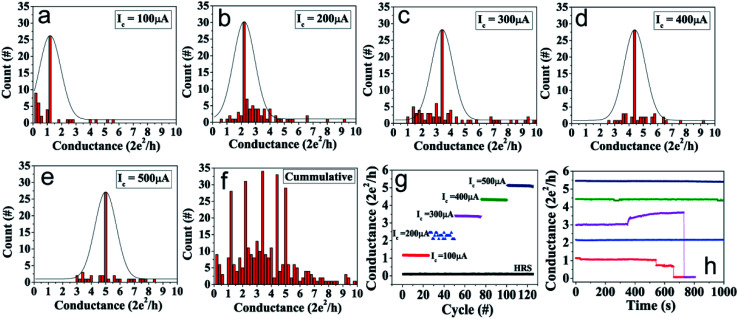
The histogram of quantized conductance values obtained during SET with *I*_c_ of (a) 100 μA (b) 200 μA, (c) 300 μA, (d) 400 μA, (e) 500 μA. Each plot shows the data of more than 60 cycles of SET for a particular *I*_c_. (f) The cumulative data of all *G*_0_ (in Fig. 2a–e) obtained for ∼300 cycles of SET. (g) The median of the distribution shows distinct conductance states after SET with current compliance of 100, 200, 300, 400, 500 μA along with HRS is exhibited for 125 switching cycles. (h) The stability of corresponding quantized conductance states shown in (g).

*Stability of QC-states & retention time*: To understand the stability of the quantized conductance states, retention time characteristics of different conductance states were studied. Different QC-states were achieved in different SET sweeps and their retention time was measured at 100 mV read voltage. Fig. 2h shows the retention time of >500 s for QC-states corresponding to 1 *G*_0_, 2 *G*_0_, 3.5 *G*_0_, 4.5 *G*_0_, and 5.5 *G*_0_. The retention time of different QC-states were observed to be increasing with increase in *G*_0_. In general, QC-states below 3 *G*_0_ were stable for less than 800 s, while the QC-states higher than 3 *G*_0_ were stable for more than 1000 s. However, on some occasions, stability over 1000 s were also observed for states <3 *G*_0_. Retention data of various other QC-states are shown in ESI Fig. S2a.† The stability of a particular QC-state depends on the strength of the corresponding conducting filament. The conducting filament diameter increases as the *G*_0_ of QC-states increase, thus making them more and more robust. The magnitude of applied read voltage during retention measurement was also found to influence the stability of QC-states (ESI Fig. S2b†).

*Inter-QC-state switching*: Once a device is switched ON to a particular QC-state, voltage sweep and current compliance conditions could be controlled to exhibit many different inter-QC-state switching in the device, be in the direction of SET (higher *G*_0_) or RESET (lower *G*_0_). Fig. 3 shows one set of four successive switching steps of inter-QC-state in SET direction of a particular device along with the corresponding QC-state retention time up to 100 s. The device was, firstly, SET to ∼2.5 *G*_0_ with *I*_c_ = 200 μA (Fig. 3a). In the subsequent voltage sweep with *I*_c_ = 300 μA, we induced an inter-QC-state switching from 2.5 *G*_0_ to ∼3 *G*_0_ state (Fig. 3b). Here, during the second sweep, the starting QC-state was found to be at 0.5 *G*_0_ instead of 2.5 *G*_0_. This change in state can be understood as instability of states below 3 *G*_0_, as discussed above. Further, the QC-state was successively switched from 3 *G*_0_ to 3.5 *G*_0_ (Fig. 3c), 3.5 *G*_0_ to 4 *G*_0_ (Fig. 3d) and 4 *G*_0_ to 4.5 *G*_0_ (Fig. 3e) during voltage sweeps with *I*_c_ = 400, 500 and 600 μA, respectively. All QC-states of the device were found to be stable for at least up to 100 s (Fig. 3h–j). The QC-states achieved during the inter-QC-state switching with *I*_c_ of 200–500 μA either matched with the peak values from the histogram of Fig. 2b–e, or fall within full-width half maxima of the peak distribution. The corresponding *I*–*V* traces of the conductance–voltage (*G*–*V*) traces shown in Fig. 3a–e are shown in the ESI Fig. S3.†

**Fig. 3:**
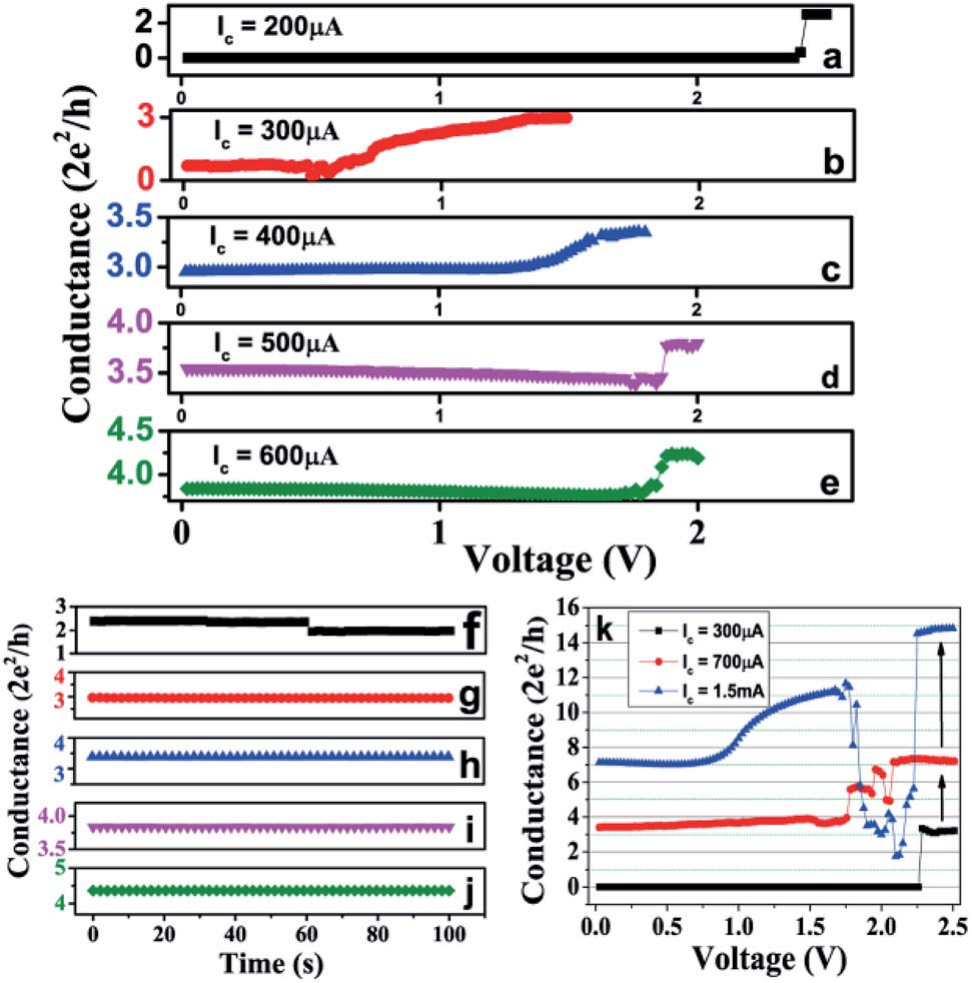
The interstate transitions between two quantized states with successive voltage sweeps during SET is exhibited in figures (a)–(e) with their corresponding final state stabilities in (f)–(j). (a) The device was first SET with *I*_c_ = 200 μA, reached to 2.5 *G*_0_. (b) In next voltage sweep with *I*_c_ = 300 μA, a state of ∼3 *G*_0_ is achieved. (c) In subsequent voltage sweep with *I*_c_ = 400 μA, state of ∼3 *G*_0_ switched to ∼3.5 *G*_0_. (d) Further increasing *I*_c_ to 500 μA, switching from ∼3.5 *G*_0_ to ∼4 *G*_0_ is induced. (e) And subsequently, in voltage sweep with *I*_c_ = 600 μA, state of 4 *G*_0_ switched to 4.5 *G*_0_. (f)–(j) Shows the stability of QC-states reached during switching steps (a)–(e). (k) The inter-QC-state switching between two quantized states with successive voltage sweeps with *I*_c_ = 300 μA, 700 μA and 1.5 mA is exhibited.

The inter-QC-state switching where the *I*_c_ values were increased in steps of 400 μA and 800 μA were also performed. In Fig. 3k, the device was switched to ∼3 *G*_0_ state with *I*_c_ = 300 μA (black trace) and then in subsequent voltage sweep with *I*_c_ = 700 μA, the device switched to ∼7 *G*_0_ state (red trace). Further, as another voltage sweep was performed with *I*_c_ = 1.5 mA, the device switched from 7 *G*_0_ to 15 *G*_0_. While switching from 3 *G*_0_ to 7 *G*_0_, the device showed indications to stop at different intermediate QC-states, however, due to higher *I*_c_ limit, the devices stopped only at 7 *G*_0_. It appears that an *I*_c_ of more than 300 μA and less than 700 μA would have possibly stabilized the device at some intermediate QC-state. During the voltage sweep with *I*_c_ = 1.5 mA, the device exhibited instability around 12 *G*_0_ state (Fig. 3k, blue trace). Since, the device can switch in both unipolar and bipolar modes, it can be understood as the device's tendency to RESET in unipolar mode due to very high currents, however, the voltage was in the range of SET (1.5–2.5 V), thus the device switched to 15 *G*_0_.

The inter-QC-state switching was also controlled and reproducibly performed in RESET direction. Fig. 4 shows three successive steps of inter-QC-state switching of a device, where different stop voltages are used to control switching to different QC levels. The device was, firstly, SET to ∼20 *G*_0_ state. Then, an inter-QC-state switching from 20 *G*_0_ to 6 *G*_0_ was induced by a voltage sweep, where −1.0 V was kept as the stop-voltage (*V*_s_), shown in Fig. 4a. In the subsequent sweeps, the QC-state switched from 6 *G*_0_ to 4.5 *G*_0_ (Fig. 4b) and from 4.5 *G*_0_ to 3.5 *G*_0_ (Fig. 4c), with *V*_s_ = −1.1 V and −1.2 V, respectively. In another subsequent sweep, the QC-state switched from 3.5 *G*_0_ to a very high resistance state (*i.e.* complete RESET) with *V*_s_ = −1.5 V, as shown in Fig. 4d. Each QC-state, after every switching, was found to be stable with time (Fig. 4e–h).

**Fig. 4:**
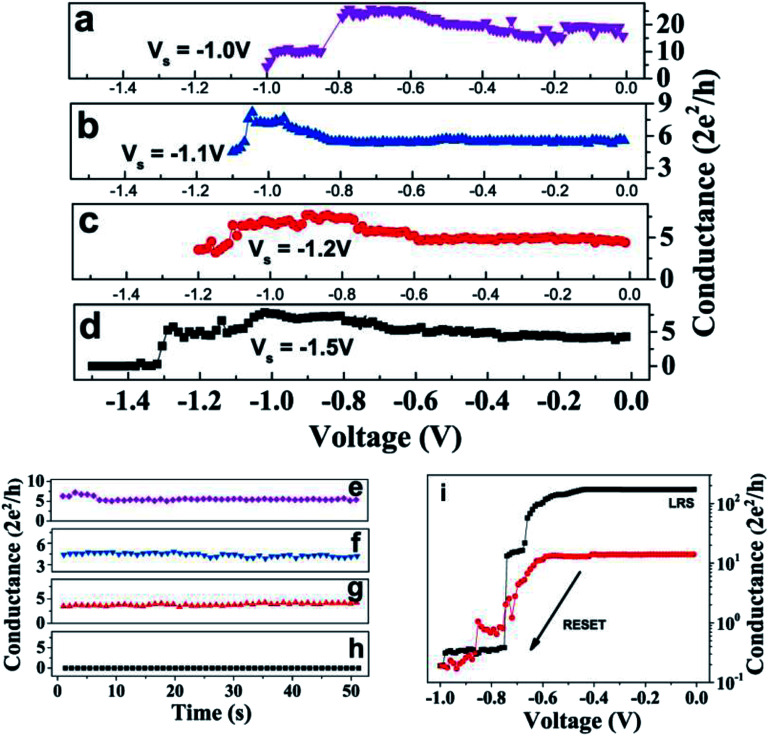
The *G*–*V* traces of inter-QC-state switching with successive voltage sweeps during RESET by varying the stop voltages. (a) The device in LRS is switched to ∼6 *G*_0_ with stop voltage of *V*_s_ = −1.0 V. (b) With of *V*_s_ = −1.1 V, a inter-QC-state switching from ∼6 *G*_0_ to ∼4.5 *G*_0_ is achieved. (c) Subsequently, during voltage sweep with *V*_s_ = −1.2 V, QC-state switched to ∼3.5 *G*_0_. (d) The figure shows a complete RESET to HRS with *V*_s_ = −1.5 V. (e)–(h) The stability with time for final QC-states achieved in figures (a)–(d) are respectively shown. (i) Complete RESET transitions are exhibited. The black trace shows the RESET from LRS to HRS and the red trace shows RESET from ∼15 *G*_0_ to HRS state. The complete SET-RESET cycles of traces in (i) is given in the ESI Fig. S5.†

During RESET switching, the critical parameter was the stop-voltage instead of the current compliance limit. For example, during the voltage sweep in Fig. 4a, the conductance starts to decrease or in other words, resistance of the device starts to increase at >−0.8 V. This voltage of −0.8 V becomes important, as, for any stop-voltage chosen little more than −0.8, the device stops at an intermediate stable QC-state, as shown in Fig. 4a–c. However, if stop-voltage is kept sufficiently high, *i.e.* close to higher end of the RESET voltage range (>−1.0 V), the device will RESET completely, as shown in Fig. 4i. However, this stop-voltage is not a fixed value, as devices have run-to run variations and have a range of voltage for RESET, as it is −0.4 V to −1.2 V for our devices. So, if a device starts to RESET at lower voltage (example: −0.6 V as shown in Fig. 4i), and the stop-voltage is chosen to be −1.0 V, the device RESETs completely earlier than −1.0 V (Fig. 4i), and thus, the device cannot be stopped at any intermediate QC-states. However, if stop-voltage would have been kept in the range −0.7 to −0.8 V for the two RESET traces in Fig. 4i, then the device could, possibly, have stopped at an intermediate QC-state.

Hundreds of inter-QC-state switching events in both SET and RESET directions were performed. The SET and RESET inter-QC-state switching (Fig. 3 and 4) are distinguished by the limiting conditions of current compliance and stop-voltage during voltage sweep cycles, respectively. However, to ensure complete RESET from any QC-state, both current compliance as well as stop-voltage needs to be kept high.

In summary, stable and reproducible QC- states were achieved in Al/Nb_2_O_5_/Pt devices by limiting current compliance during the current–voltage measurements. All the states were stable at least for 500 s, and the higher conductance states exhibited longer retention times. The stable quantized states could be controllably switched to higher *G*_0_ (SET direction) or to lower *G*_0_ (RESET direction) states, by imposing the current compliance or stop-voltage limits, respectively. The conditions for complete RESET, starting from any quantized state, could also be selectively induced by lifting limiting conditions on current or voltage during RESET voltage sweep. The possibility of utilizing the QC-states in the resistive devices for multilevel logic shows potentials for achieving high-density storage.

## Conflicts of interest

The authors declare no competing financial interest.

## Supplementary Material

RA-009-C9RA00726A-s001
