# Supplementary material for: Controlled inter-state switching between quantized conductance states in resistive devices for multilevel memory
Source: RSC Adv. 2019 Mar 25;9(17):9494–9. doi: 10.1039/c9ra00726a (PMC9062199; doi:10.1039/c9ra00726a)
Supplement: RA-009-C9RA00726A-s001 [file RA-009-C9RA00726A-s001.pdf]

Electronic Supplementary Material (ESI) for RSC Advances.
This journal is © The Royal Society of Chemistry 2019

Supplementary information

Controlled inter-state switching between quantized conductance states in resistive devices for multilevel memory

Sweety Deswal,[†,‡] Rupali R. Malode,[§] Ashok Kumar,[†,‡] and Ajeet Kumar*,[†,‡]

[†]Academy of Scientific and Innovative Research (AcSIR), Ghaziabad- 201002, India.

[‡]CSIR-National Physical Laboratory, Dr. K.S. Krishnan Marg, New Delhi 110012, India.

[§]Maulana Azad National Institute of Technology, Bhopal, Madhya Pradesh 462003, India.

Methods

The Al/Nb_2O_5/Pt based resistive switching devices were fabricated by depositing ~30 nm Nb_2O_5 thin films over Platinised Si/SiO_2 substrate and subsequently depositing Al as top electrode. After standard cleaning of the substrate, the bottom electrode was masked with NiCr alloy strip of width ~0.7 mm to make Pt available for bottom electrode contact. Then, Nb_2O_5 thin films were grown using reactive dc magnetron sputtering at a pressure of 2.0×10^{-2} mbar in gas mixture of Ar (94%) and O_2 (6%), while the base pressure was evacuated at ~6 $\times10^{-7}$ mbar. Thereafter, the top electrode of Al was deposited with areas ranging from 100 to 500 μm^2 using a shadow mask by thermal evaporation technique. The current-voltage (I-V) characteristics were measured on two probe station with Agilent 2450 source-measure unit. All the measurements were performed at room temperature by applying the voltage source on the Al top electrode with the Pt bottom electrode grounded. The voltage was swept keeping a current compliance for all electrical measurements. This method of fabricating device and electrical measurement was also followed in our previous report.[s1]

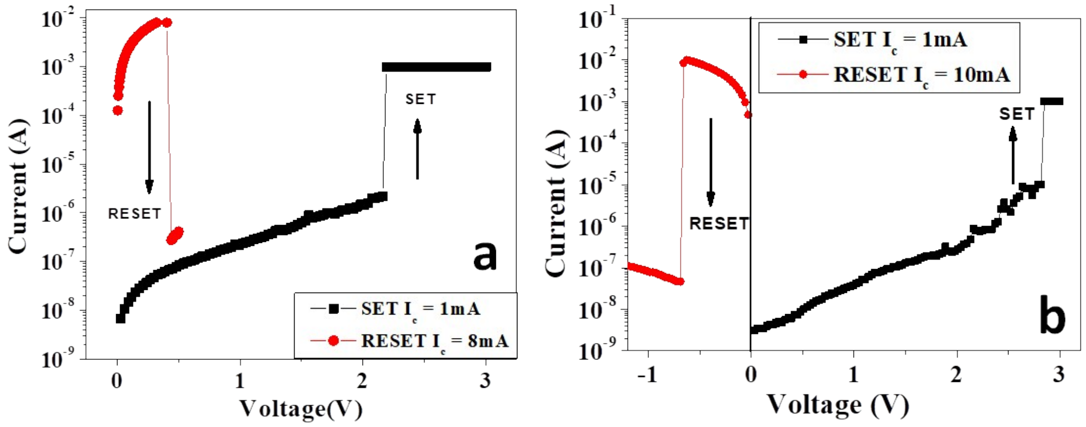

Fig. S1 The Al/Nb_2O_5/Pt device shows both unipolar (a) and bipolar (b) switching characteristics. Both (a) and (b) are consecutive switching cycles of one device. Once the electroformation is done, device can switch in both modes. After the SET voltage step with I_c = 1 mA, the RESET step can be taken in either polarity of the voltage. The current required for the bipolar switching (10 mA) is similar to unipolar switching (8 mA). This is similar to previous reported observations of resistive devices showing both unipolar and bipolar switching characteristics.[s2]

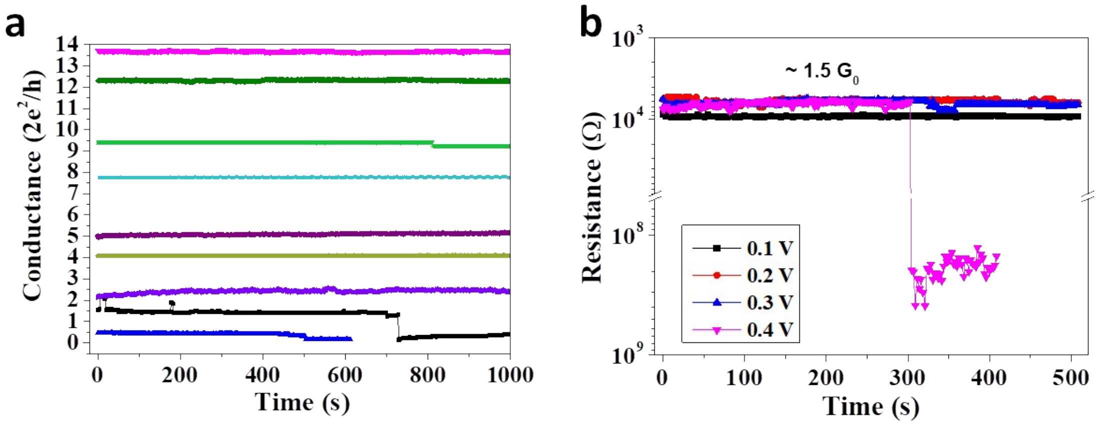

Fig. S2 (a) The retention time of various quantized states observed during SET, is exhibited. The states below 3 G_0 showed stablility for ~ 500 to 750 seconds. The weaker filament formed with smaller current compliance, results in lower stability of the states. The states ≥ 3 G_0 showed stability for at-least 1000 seconds. This higher stability is due to formation of stronger filament with larger current compliance values. (b) shows resistance of a QC-state of 1.5 G_0 with four different read voltages. The 1.5 G_0 QC-state was stable for longer time at low read voltages, up to 0.3 V, and became unstable at 0.4 V.

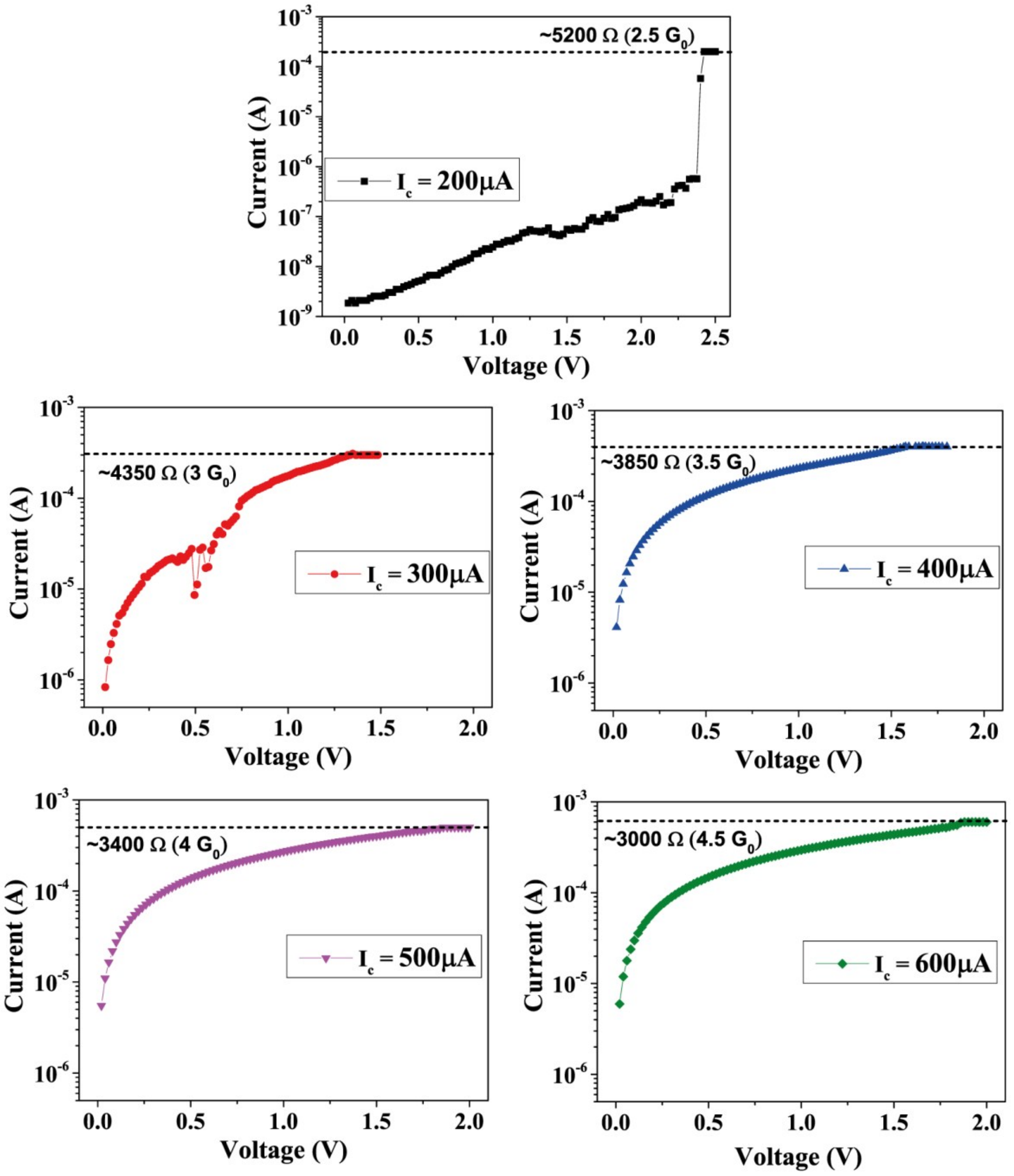

Fig. S3 The *I-V* traces of inter-quantized state switching in the SET direction with I_c = 200, 300, 400, 500, and 600 �A is shown in (a)-(e), respectively. The corresponding *G-V* traces of these curves are presented in the manuscript in Fig. 3a-e.

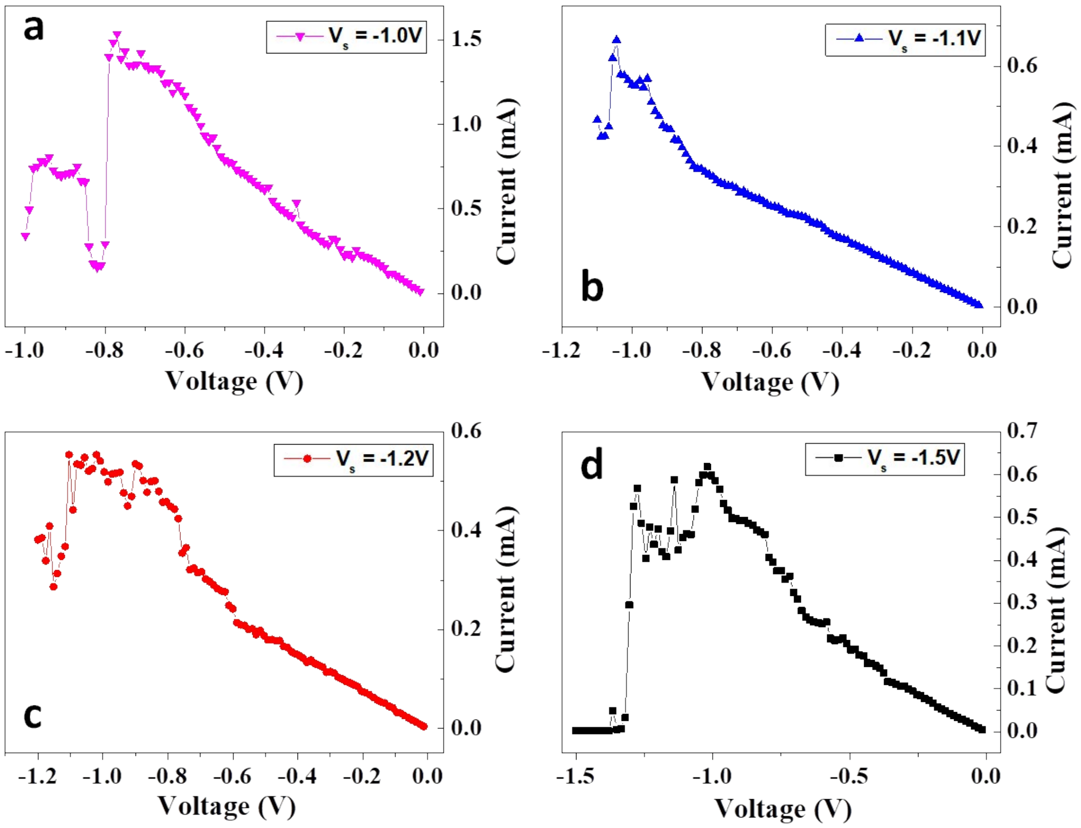

Fig. S4 ***I-V*** **traces of interstate switchings between quantized states with successive voltage sweeps during RESET are exhibited by varying the magnitude of stop voltages. (a)-(d) show four RESET traces with V_s = -1.0, -1.1, -1.2, and -1.5 V. The corresponding** ***G-V*** **traces are presented in the manuscript in the Fig. 4a-d.**

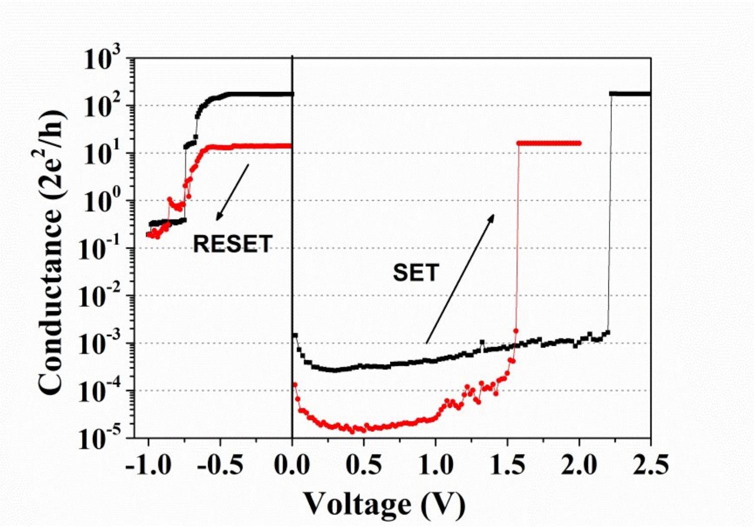

Fig. S5 Complete SET-RESET cycles of the traces presented in the Fig. 4i.

References

S1 S. Deswal, A. Kumar and A. Kumar, *AIP Adv.*, 2018, **8**, 085014.

S2 Y. Sharma, S. P. Pavunny, E. Fachini, J. F. Scott, R. S. Katiyar, *J. Appl. phys.* 2015, **118**, 094506.
